# Continuous Fentanyl Background Infusion Regimen Optimised by Patient-Controlled Analgesia for Acute Postoperative Pain Management: A Randomised Controlled Trial

**DOI:** 10.3390/jcm9010211

**Published:** 2020-01-13

**Authors:** Jihoon Hwang, Sang Kee Min, Yun Jeong Chae, Gang Mee Lim, Han Bum Joe

**Affiliations:** Department of Anesthesiology and Pain Medicine, Ajou University School of Medicine, 164, Worldcup-ro, Yeongtong-gu, Suwon 16499, Korea; kironojh@gmail.com (J.H.); anesmin@nate.com (S.K.M.); yjchae06@hotmail.com (Y.J.C.); limjks7@naver.com (G.M.L.)

**Keywords:** postoperative pain management, fentanyl, patient-controlled analgesia, background infusion

## Abstract

Owing to a lack of studies investigating the effect of adjustments in fentanyl background infusion (BI) with patient-controlled analgesia (PCA) on postoperative analgesia, we evaluated three BI regimens with fentanyl PCA for acute postoperative pain management. This randomised controlled trial enrolled 105 patients, who were assigned to three parallel groups: constant rate BI of 2 mL/h (CRBI group); time-scheduled decremental BI of 6, 2 and 1 mL/h (TDBI group); and BI rates optimised to the demand of PCA (POBI group). The incidence of insufficient analgesia, visual analogue scale (VAS) pain score and side effects were evaluated. The incidence of insufficient analgesia in the post-anaesthesia care unit was lower in the TDBI and POBI groups than the CRBI group. Incidence of insufficient analgesia in the ward was lower in the POBI group than the CRBI group. Postoperative VAS scores were significantly lower in the TDBI and POBI groups for up to 4 h and 24 h, respectively, compared with the CRBI group. Side effects and infused fentanyl dose were highest in the CRBI group. Adjusting BI rate based on time or patient demands could improve postoperative analgesia and reduce side effects. Compared to a constant BI rate, PCA-optimised BI achieved higher patient satisfaction.

## 1. Introduction

Acute postoperative pain management is essential for patient care post-surgery and is associated with haemodynamic stability, rapid improvement of respiratory function and rapid recovery of patients [1,2]. However, postoperative pain management remains a challenge despite medical advances.

Intravenous fentanyl is commonly used for controlling acute postoperative pain [3,4] by patient-controlled analgesia (PCA) with continuous background infusion (BI) [5,6]. This maintains basal fentanyl infusion, with immediate self-administration of analgesics according to each patient’s requirements. Despite this, acute postoperative pain control can be inadequate during the initial postoperative period [7,8,9] due to various factors, including the pharmacokinetic properties of fentanyl. When fentanyl is infused at a constant rate within the recommended regimens (<1.1 μg/kg/h) [6,10,11,12,13,14], the effect-site concentration (C_eff_) of fentanyl shows a delayed increase until a steady-state of the C_eff_ is reached, despite PCA demand-doses [15]. Therefore, PCA with time-scheduled decremental BI rates (TDBI), which initially increases and then gradually decreases the BI rate seems more effective than PCA with a constant rate BI (CRBI) [15].

However, postoperative pain and analgesic requirements vary among patients [16,17], and fentanyl shows a wide inter-individual range, with the minimum effective plasma concentration (C_p_) of 0.63 ng/mL ranging from 0.23 to 1.18 ng/mL [10]. Therefore, to reflect these variations, we designed a PCA-optimised BI (POBI) to provide adequate fentanyl dose for postoperative pain and reduce the incidence of side effects, using an electronic drug infuser with a program for controlling BI rate according to patient’s PCA demands. In this study, we evaluated the fentanyl PCA with TDBI and POBI in comparison to that with CRBI for postoperative pain control after total laparoscopic hysterectomy. In this study, we evaluated TDBI and POBI in comparison with CRBI in a continuous infusion method of fentanyl PCA for postoperative pain control after total laparoscopic hysterectomy.

## 2. Methods

### 2.1. Study Design and Setting

This was a single-centre, prospective, double-blind, randomised controlled trial with three parallel groups carried out at Ajou University Hospital (Suwon, South Korea) from December 2016 to September 2017. The trial was approved by the institutional review board of Ajou University Hospital (AJIRB-MED-OBS-16-106) and was registered at CRIS registration (KCT0002156). Written informed consent was obtained from all patients.

### 2.2. Subjects

Eligible participants were female patients of American Society of Anesthesiologists physical status 1–2, aged 20–65 years, who wanted postoperative pain management with PCA after total laparoscopic hysterectomy under general anaesthesia. Patients with a history of neurologic, psychiatric, endocrinologic, renal or hepatic disorders, those with a history of drug or alcohol abuse and patients with chronic pain diseases were excluded. Patients weighing more or less than 20% of the ideal body weight were also excluded. Patients who received opioids or analgesics, except remifentanil during anaesthesia, showed delayed recovery of consciousness at more than 30 min after the administration of anaesthetics was stopped following completion of surgery, developed complications requiring treatment or required postoperative intensive care unit management were also excluded. On the day before surgery, patients were informed about acute pain management guidelines, use of PCA infuser, expressing the degree of pain and possible complications and treatments.

### 2.3. Anaesthesia

All patients underwent standardised total intravenous anaesthesia. Glycopyrrolate 0.2 mg, with no sedatives or analgesics, was administered intravenously as premedication. Routine monitoring consisted of electrocardiograph, pulse oximetry, non-invasive arterial blood pressure, end-tidal carbon dioxide partial pressure and bispectral index (BIS). Total intravenous anaesthesia was induced using propofol (Fresofol, Fresenius Kabi, Homburg, Germany) and remifentanil (Ultiva, GlaxoSmithKline, Rixensart, Belgium) target-controlled infusions were administered using a two-channel infusion pump (Orchestra, Fresenius Vial, Brezins, France) with corresponding pharmacokinetic/pharmacodynamic (PK/PD) models [18,19]. For neuromuscular blockade, rocuronium 0.6 mg/kg was administered intravenously. After tracheal intubation, propofol and remifentanil target-controlled infusions were titrated to maintain BIS between 40 and 60 and systolic blood pressure and heart rate ±20% of baseline values. Palonosetron 0.075 mg was administered intravenously after the induction of anaesthesia to prevent postoperative nausea and vomiting (PONV) [20,21]. When skin closure began, propofol and remifentanil infusion was stopped. Thereafter, when the C_eff_ of remifentanil target-controlled infusion decreased to 1.0 ng/mL, fentanyl (Fentanyl citrate, HanaPharm. Co., Republic of Korea) 0.5 μg/kg was injected intravenously over 30 s [22]. Residual neuromuscular blockade was reversed with glycopyrrolate 0.40 μg/kg and pyridostigmine 0.2 mg/kg. When patients opened their eyes, deep breathing was encouraged, and extubation was performed. The patient was then transported to the post-anaesthesia care unit (PACU). The entire anaesthesia procedure and the PCA preparation were performed by an anaesthesiologist otherwise not involved in the postoperative pain assessment.

### 2.4. Study Interventions

A programmable PCA infuser (PS-1000, Unimedics Corp., Ltd., Seoul, Korea) filled with 100 mL of fentanyl diluent was prepared, and each fentanyl diluent was prepared according to body weight of 0.25 μg/kg per 1.0 mL of diluent, using normal saline [14]. The infuser was connected at the proximal portion of the patient’s indwelling cannula using a three-way stopcock, and an anti-reflux one-way valve was inserted to prevent the back-flow to the gravity infusion line for anticipated occlusions.

Blinded patients were randomly allocated in a 1:1:1 ratio to one of the three (CRBI, TDBI and POBI) groups, with 35 patients per group, using the Excel ‘Random’ function (Microsoft Office Excel 2010). The allocation process was conducted by a colleague not involved in this project. The randomisation results were concealed within serially numbered opaque envelopes, which were opened before induction of anaesthesia.

In the CRBI group, the BI was maintained at a constant rate of 2.0 mL/h (0.5 μg/kg/h) for 24 h postoperatively. In the TDBI group, the BI rate was maintained at 6.0 mL/h (1.5 μg/kg/h) for 1 h postoperatively, then decreased to 2.0 mL/h (0.5 μg/kg/h) between 1–8 h followed by 1.0 mL/h (0.25 μg/kg/h) between 8–24 h. In the POBI group, using the programmable mode embedded in the infuser, the administration of fentanyl diluent started at 6.0 mL/h (1.5 μg/kg/h). When the PCA mode was activated by the patient, the BI rate automatically increased by 0.5 mL/h. When there was no PCA demand for 30 min, the BI rate decreased by 1.0 mL/h. The maximum permissible BI rate was 8.5, 6 and 3 mL/h for 0–2, 2–8 and 8–24 h, respectively, to prevent excessive rise of fentanyl C_eff_. The minimum permissible infusion rate was 2 and 1 mL/h for 0–8 and 8–24 h, respectively, to maintain minimum effective analgesic concentration. PCA demand-dose was 1.0 mL (0.25 μg/kg) with a 15-min lock-out time in all three groups.

### 2.5. Assessment of Outcomes

Non-invasive arterial blood pressure, oxygen saturation, heart rate and respiratory rate were monitored and the degree of pain was assessed in the PACU at 15-min intervals. Patients were asked to express the degree of pain using 10 cm visual analogue scale (VAS) at rest (‘no pain = 0 cm’ to ’the worst pain imaginable = 10 cm’).

The primary outcome of this study was ‘insufficient analgesia’ during the first postoperative hour in the PACU, defined by one or more episodes of ‘VAS > 4′ at every 15 min-interval assessment. When a patient showed insufficient analgesia, fentanyl diluent 1 mL (0.25 μg/kg) was administered using PCA module; if insufficient analgesia persisted for longer than 15 min, intravenous ketorolac (Tarasyn, Roche Korea Co., Ltd., Seoul, Republic of Korea) 30 mg was given as a rescue analgesic.

After 1 h, patients who met the PACU discharge criteria were transported to the general ward. Patients were instructed to use PCA voluntarily in the ward. If the pain persisted despite the use of PCA and the patient required additional analgesics, intravenous ketorolac 30 mg was also administered as a rescue analgesic. If the patient experienced severe unbearable side effects, the PCA would be suspended temporarily or stopped as per patient’s request.

During the pain management period, data such as change in PCA infusion rate, usage of PCA button, and real-time dose and cumulative dose were collected at 30 s intervals for analysis.

The secondary outcome was assessment of analgesia and patient conditions in the ward at postoperative time points 2, 3, 4, 6, 12 and 24 h. The assessor, blinded to the group allocation, recorded the pain score and side effects such as nausea, vomiting, headache, dizziness and itching. Insufficient analgesia in the ward was also defined by one or more episodes of ‘VAS >4′. If rescue analgesics were administered, the frequency and dose were also recorded. For severe complaints of PONV, palonosetron 0.025 mg was administered intravenously.

At the time of withdrawal from the study, or 24 h postoperatively, the satisfaction score for overall postoperative pain management (based on an eleven-point categorical scale of ‘0 = worst’ to ‘10 = best’) was recorded for each patient; patients were questioned for their reasoning in case of dissatisfaction.

### 2.6. Simulation of C_eff_

We simulated the predicted C_eff_ of fentanyl of the three groups using the PK/PD software (STANPUMP, SL Shafer, Department of Anesthesia, Stanford University, USA) with the weight-scaled three-compartment PK model [23] and the effect-site compartment [24,25].

### 2.7. Sample Size and Statistics

According to a previous study [15], the incidence of insufficient analgesia in the PACU was 79% with 0.5 μg/kg/h of BI and 48% with time-scheduled decremental BI. Under the assumption that the incidence of insufficient analgesia in the TDBI and POBI groups differ from the CRBI group at least to a similar extent, a sample size of 105 achieved 80% power using 2 degrees of freedom Chi-square test with a significance level of 0.05.

Statistical analysis was performed using SPSS version 21 (SPSS Inc., Chicago, IL, USA). All analyses followed the intention-to-treat principle. Data were expressed as mean with standard deviation (SD), median with interquartile range (IQR) or number of patients (%). Continuous data were tested for normality of distribution using the Kolmogorov-Smirnov test. Normally distributed data were analysed using one-way analysis of variance (ANOVA). Non-normally distributed data were compared between groups using Kruskal-Wallis test. Categorical data were analysed with a Chi-square test or Fisher’s exact test. Repeated-measures ANOVA was used to compare VAS changes over time among the groups, followed by Tukey’s post-hoc test. A *P-*value < 0.05 was considered statistically significant. Bonferroni post-hoc test was used for multiple comparison between groups and a Bonferroni-adjusted *P*-value < 0.017 (0.05/3) was considered statistically significant. The primary outcome of each group was reported with 95% confidence interval (CI).

## 3. Results

**Figure 1:**
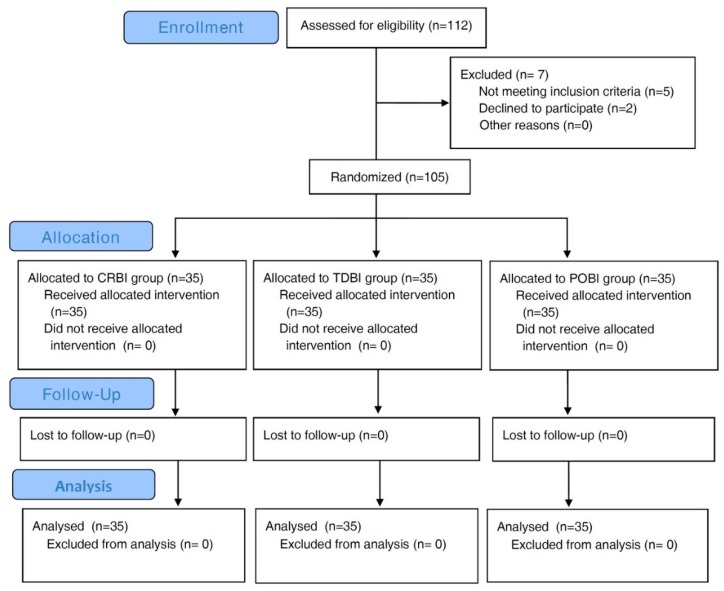
Consort flow diagram of recruitment and assessment of study participants. CRBI group, fentanyl patient-controlled analgesia (PCA) with constant rate background infusion (BI); TDBI group, fentanyl PCA with time-scheduled decremental BI; POBI group, fentanyl PCA with PCA-optimised BI.

In total, 112 female patients were screened, of which two refused participation, and five were excluded because they did not meet the inclusion criteria. The participant flow diagram for the study is presented in Figure 1. Finally, 105 patients were enrolled, and all patients completed the study. Patient characteristics are presented in Table 1. There was no statistically significant difference among the groups.

At the PACU, the incidence of insufficient analgesia was significantly lower in the TDBI (68.6%, 95% CI: 52.0–81.5; *P =* 0.012) and POBI groups (51.4%, 95% CI: 35.6–67.0; *P* < 0.001) compared to the CRBI group (94.3%, 95% CI: 81.4–98.4). At ward, the incidence of insufficient analgesia was significantly lower in the POBI group only, compared to the CRBI group (*P* < 0.001). PCA demand was greater in the CRBI group than in the POBI group (*P* = 0.002). A higher number of patients required rescue analgesics in the TDBI group than in the POBI group (*P* = 0.012). Patients who suspended PCA were not significantly different among groups (Table 2).

**Figure 2:**
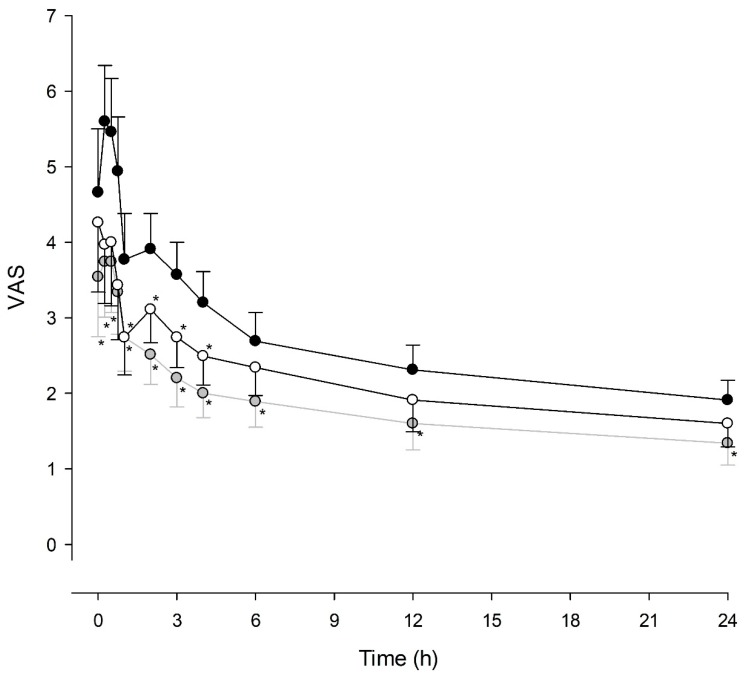
Time-courses of the visual analogue pain rating scale (VAS) scores during the postoperative analgesia. CRBI group (black circle), fentanyl patient-controlled analgesia (PCA) with constant rate background infusion (BI); TDBI group (white circle), fentanyl PCA with time-scheduled decremental BI; POBI group (grey circle). The circles and whiskers indicate means and 95% CI. * *P* < 0.05 compared with CRBI group.

The VAS scores for postoperative pain 24 h post-surgery are presented in Figure 2, which significantly differed among the three groups over the 11 time points (group effect, *P* < 0.001). The VAS score was significantly lower in TDBI and POBI groups than in the CRBI group (TDBI versus CRBI group, *P* = 0.008; POBI versus CRBI group, *P* < 0.001). Multiple comparison between groups showed significantly lower VAS scores in the POBI group up to 24 h and in the TDBI group up to 4 h postoperatively than in the CRBI group (Figure 2). The incidence of PONV and headache were not different among groups. The incidence of itching and dizziness in the CRBI group was higher than in TDBI (*P* < 0.001 for itching, *P* = 0.004 for dizziness) and POBI (*P* = 0.003 for itching, *P* = 0.003 for dizziness) groups. Patient satisfaction score was significantly higher in the POBI group than in CRBI group (*P* = 0.005). PONV was the most common reason for dissatisfaction in all three groups, with 60.0, 45.7 and 37.1% in the CRBI, TDBI and POBI groups, respectively.

**Figure 3:**
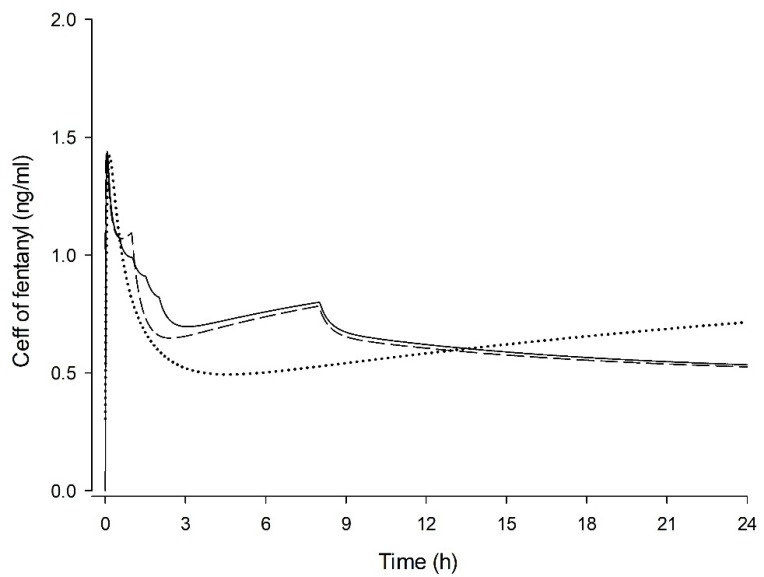
Time-courses of predicted effect-site concentration (C_eff_) of fentanyl with the three background infusions (BI) without patient-controlled analgesia (PCA) demand-dose after intravenous injection of fentanyl 0.5 μg/kg at time 0 h. CRBI group (dotted line), fentanyl PCA with constant rate BI; TDBI group (dashed line), fentanyl PCA with time-scheduled decremental BI; POBI group (solid line), fentanyl PCA with PCA-optimised BI.

**Figure 4:**
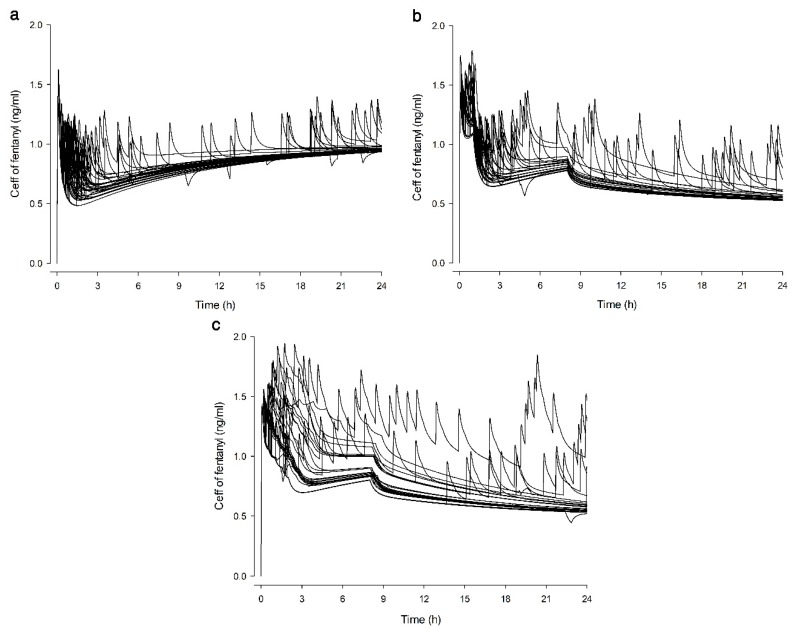
Time-courses of predicted effect-site concentration (C_eff_) of fentanyl based on the infusion data stored in patient-controlled analgesia (PCA) infusers of actual patients in each group. Each peak represents the increased fentanyl C_eff_ by PCA demand-dose. (**a**) CRBI group, PCA with constant rate background infusion (BI). (**b**) TDBI group, fentanyl PCA with time-scheduled decremental BI. (**c**) POBI group, fentanyl PCA with PCA-optimised BI.

The predicted fentanyl C_eff_ of each group when only BI was injected without PCA demand dose is shown in Figure 3. The predicted C_eff_ of fentanyl in TDBI and POBI groups was higher up to 13 h postoperatively compared to the CRBI group (Figure 3). The predicted fentanyl C_eff_ of each group based on the data stored in PCA infusers of patients are shown in Figure 4. The difference in predicted C_eff_ appeared to be greater in the POBI group (Figure 4c) than in the other groups (Figure 4a,b).

## 4. Discussion

This study demonstrated that the incidence of insufficient analgesia was significantly lower in TDBI and POBI groups than in the CRBI group during the first postoperative hour. After this, the incidence of insufficient analgesia was lower in only the POBI group than in CRBI group. The postoperative pain scores were significantly lower using TDBI (up to 4 h) and POBI (up to 24 h) than in CRBI. Total infused fentanyl dose was higher and side effects such as itching and dizziness were more common in the CRBI group. Patient satisfaction scores were higher in the POBI than in CRBI group. Respiratory depression was absent in all groups.

The conventional PCA with BI is an individualised infusion method allowing patients to immediately titrate the opioid dose corresponding to their analgesia needs. This leads to improved postoperative analgesia and patient satisfaction compared with non-patient-controlled intermittent parenteral analgesia [26]. However, due to concerns of adverse effects and overdose, reduced PCA demand-dose and increased lock-out intervals tend to be implemented, which can lead to inadequate analgesia [7,8,9]. Some evidence suggests that using BI could increase the risk of respiratory depression [27]. Lowering the infusion rates under recommended regimens (<1.1 μg/kg/h) [10,11,12,13,14] may cause a delay in reaching the desired analgesia concentration. Conversely, increasing the BI rate over recommended regimens increases the predicted fentanyl C_eff_ and accumulation over time, thereby increasing the risk of respiratory depression [15,28]. Although the fentanyl C_eff_ could be increased temporarily by PCA demand-dose while maintaining the recommended infusion rate during the early postoperative period, the peripheral redistribution of fentanyl may soon cause a low C_eff_ [29], contributing to insufficient analgesia during the lock-out time set for safety. Unlike a conventional PCA infuser where BI rate cannot be changed automatically, the new PCA device with a program allows the adjustment of BI rates over time. Therefore, TDBI using this device could meet the initial therapeutic range for analgesia and reduce the accumulation of fentanyl over time. Furthermore, other studies have shown that the fentanyl or morphine PCA with TDBI provided more effective analgesia without increasing side effects compared to CRBI [15,30].

With POBI, the BI rate varies according to the patient’s PCA demands in order to reflect differences in analgesic requirements among individuals, unlike with TDBI. In the PK/PD simulation of fentanyl, the difference in predicted C_eff_ appeared to be greater within the POBI group (Figure 4c). This shows that PCA with POBI reflects patient-to-patient differences better than does PCA with the TDBI and CRBI. Our results indicate that the fentanyl PCA with POBI might be more effective than PCA with TDBI with respect to analgesia, as the POBI group had lower incidences of insufficient analgesia and lower VAS scores for a longer time period. The POBI group also showed reduced need for rescue analgesics compared with the TDBI group and higher patient satisfaction compared with the CRBI group. However, postoperative pain score and side effects did not differ significantly between POBI and TDBI groups.

The 50% depression of the slope of the ventilation-CO_2_ response curve in C_p_ of fentanyl is between 2.0–3.1 ng/mL [31], while the minimum effective analgesic C_p_ ranges from 0.23 to 1.18 ng/mL [10]. In this study, the predicted C_eff_ of fentanyl was lower than 2.0 ng/mL in all patients, and no patient showed respiratory depression. Previous studies showed no differences in side effects between conventional CRBI and TDBI [15,30]. However, itching and dizziness were less frequent in TDBI and POBI groups possibly due to the lower total infused fentanyl dose for 24 h post-surgery (Table 2) [26,32]. In all three groups, PONV was the main cause of dissatisfaction and discontinuation of PCA. With the increasing use of antiemetics, adjustment of POBI settings and non-opioid multimodal analgesic treatment should be considered. Although no serious side effects occurred, whether POBI is indeed safe requires collection of prospective safety data from a significantly larger group. In addition, it is important to appropriately set an upper BI limit to prevent adverse effects from occurring.

There are several limitations to this study. First, this study was limited to female patients and cannot be generalised to patients of other demographics and clinical characteristics such as degree of postoperative pain. However, since the POBI is more individualised, adequate analgesia using fentanyl PCA with POBI can also be expected under other circumstances. Second, the sample size in this study was insufficient to determine differences between TDBI and POBI. Therefore, further studies with larger sample sizes can demonstrate more evident benefits of POBI over TDBI. Third, this study only assessed early postoperative pain up to 24 h. Long-term postoperative satisfaction, treatment and morbidity due to chronic pain were not evaluated.

In conclusion, fentanyl PCA with adjusted BI rates over time (TDBI) or according to the patient’s PCA demands (POBI) improved postoperative analgesia, reduced side effects and had lower total infused fentanyl doses compared with PCA with CRBI, which had a higher incidence of inadequate analgesia, increased side effects and a higher total infused fentanyl dose. PCA with POBI achieved the best results. Therefore, PCA with POBI would be an effective analgesic method similar to PCA with TDBI, that reflects inter-individual differences in postoperative pain and analgesic requirements. The effectiveness and safety of POBI in patients with various surgical procedures should be verified, and appropriate protocols of POBI should be further studied.

## Figures and Tables

**Table 1:** Characteristics of patients receiving post-operative anaesthesia, and duration of surgery and anaesthesia. Data are represented as mean ± standard deviation.

	CRBI Group (*n* = 35)	TDBI Group (*n* = 35)	POBI Group (*n* = 35)
Age (years)	46.7 ± 4.0	47.9 ± 7.3	48.5 ± 9.4
Weight (kg)	60.2 ± 7.0	61.0 ± 8.9	60.0 ± 9.2
Height (cm)	159.5 ± 4.1	158.3 ± 5.6	157.7 ± 5.8
Duration of surgery (min)	98.0 ± 34.1	88.5 ± 35.0	85.1 ± 46.5
Duration of anaesthesia (min)	128.0 ± 34.3	118.3 ± 37.5	116.7 ± 53.7

Not significant among groups CRBI group, fentanyl patient-controlled analgesia (PCA) with constant rate background infusion (BI); TDBI group, fentanyl PCA with time-scheduled decremental BI; POBI group, fentanyl PCA with PCA-optimised BI.

**Table 2:** Postoperative analgesic profiles, side effects and patient satisfaction scores after receiving post-operative anaesthesia. Data are represented as number (%), or median (interquartile range).

	CRBI Group (*n* = 35)	TDBI Group (*n* = 35)	POBI Group (*n* = 35)	*P*-Value
Insufficient analgesia				
At PACU	33 (94.3)	24 (68.6) ^a^	18 (51.4) ^b^	**<0.001**
At Ward	14 (40.0)	5 (14.3)	1 (2.9) ^b^	**<0.001**
Total fentanyl dose (μg)	722.5 (632.7–815.6)	600.6 (508.8–671.6) ^a^	587.1 (490.2–667.7) ^b^	**0.002**
Number of PCA demands	4.0 (2.8–5.0)	3.0 (1.0–6.0)	1.0 (1.0–4.0) ^b^	**0.009**
Number of Rescue analgesics	2.0 (1.0–3.0)	2.0 (1.0–3.0)	2.0 (0.0–3.0) ^c^	**0.027**
Cessation of PCA	8 (22.9)	5 (14.3)	7 (20.0)	0.649
Side effects				
PONV	23 (65.7)	17 (48.6)	15 (42.9)	0.137
Itching	11 (31.4)	0 (0.0) ^a^	1 (2.9) ^b^	**<0.001**
Headache	10 (28.6)	6 (17.1)	4 (11.4)	0.177
Dizziness	30 (85.7)	21 (60) ^a^	18 (51.4) ^b^	**0.007**
Patient satisfaction (0–10)	8 (5–9)	9 (7–10)	9 (8–10) ^b^	**0.013**

Categorical data (insufficient analgesia, cessation of PCA, and side effects) were analysed with a Chi-square test or Fisher’s exact test with Bonferroni correction. Non-normally distributed data (total fentanyl dose, number of PCA demands, number of rescue analgesics and patient satisfaction) were compared using Kruskal-Wallis test and followed by Mann-Whitney U-test with Bonferroni correction. ^a,b,c^ Bonferroni-adjusted *P*-value < 0.017 for multiple comparison between groups in post-hoc test (^a^ Between CRBI and TDBI group, ^b^ Between CRBI and POBI group, ^c^ Between TDBI and POBI group).CRBI group, fentanyl patient-controlled analgesia (PCA) with constant rate background infusion (BI); TDBI group, fentanyl PCA with time-scheduled decremental BI; POBI group, fentanyl PCA with PCA-optimised BI; PACU, post-anaesthesia care unit; PONV, postoperative nausea and vomiting. *P*-values in bold indicate statistical significance < 0.05.
